# Good practices for dialysis education, treatment, and eHealth: A scoping review

**DOI:** 10.1371/journal.pone.0255734

**Published:** 2021-08-11

**Authors:** Anita van Eck van der Sluijs, Sanne Vonk, Brigit C. van Jaarsveld, Anna A. Bonenkamp, Alferso C. Abrahams

**Affiliations:** 1 Department of Nephrology and Hypertension, University Medical Center Utrecht, Utrecht, The Netherlands; 2 Department of Nephrology, Amsterdam University Medical Centers, Location AMC, Amsterdam, The Netherlands; 3 Diapriva Dialysis Center, Amsterdam, The Netherlands; Mayo Clinic Minnesota, UNITED STATES

## Abstract

**Background:**

Recommendations regarding dialysis education and treatment are provided in various (inter)national guidelines, which should ensure that these are applied uniformly in nephrology and dialysis centers. However, there is much practice variation which could be explained by good practices: practices developed by local health care professionals, which are not evidence-based. Because an overview of good practices is lacking, we performed a scoping review to identify and summarize the available good practices for dialysis education, treatment, and eHealth.

**Methods:**

Embase, Pubmed, the Cochrane Library, CINAHL databases and Web of Science were searched for relevant articles using all synonyms for the words ‘kidney failure’, ‘dialysis’, and ‘good practice’. Relevant articles were structured according to the categories dialysis education, dialysis treatment or eHealth, and assessed for content and results.

**Results:**

Nineteen articles (12 for dialysis education, 3 for dialysis treatment, 4 for eHealth) are identified. The good practices for education endorse the importance of providing complete and objective predialysis education, assisting peritoneal dialysis (PD) patients in adequately performing PD, educating hemodialysis (HD) patients on self-management, and talking with dialysis patients about their prognosis. The good practices for dialysis treatment focus mainly on dialysis access devices and general quality improvement of dialysis care. Finally, eHealth is useful for HD and PD and affects both quality of care and health-related quality of life.

**Conclusion:**

Our scoping review identifies 19 articles describing good practices and their results for dialysis education, dialysis treatment, and eHealth. These good practices could be valuable in addition to guidelines for increasing shared-decision making in predialysis education, using patients’ contribution in the implementation of their dialysis treatment, and advanced care planning.

## Introduction

According to the latest estimates, more than 320 million patients are treated with dialysis worldwide [1]. In most developed countries, patients start dialysis after having received education on different treatment options (i.e. dialysis, transplantation, and conservative care) [2–4]. Recommendations regarding education and dialysis treatment are given in various (inter)national guidelines [5–10]. These, preferably evidence-based, recommendations assist health care professionals in the guidance and treatment of chronic kidney disease (CKD) patients in order to provide the best possible care.

Guidelines should ensure that complete and objective education is provided to CKD patients about all treatment options [5]. In addition, guidelines should assure that practical execution of a specific dialysis treatment (i.e. hemodialysis (HD) or peritoneal dialysis (PD)) is more or less the same in all centers. However, this does not always seem to be the case. In 2010, it was shown that variation in center-specific factors (e.g. number of patients, in-center HD treatment capacity, and availability of a late dialysis shift) in the United States influenced the utilization of home dialysis (i.e. home HD and PD) [11]. This also appears to be true for many other countries when looking at the variation in PD utilization [12]. In addition, practice variation *within* a country seems to associate with a broad range in the percentage of dialysis patients treated with home dialysis [13]. Probably part of this variation can be explained by so-called ‘good practices’ which are developed locally.

The term ‘good practice’, also referred to as ‘best practice’, denotes ‘…*a practice that has been proven to work well and produce good results*, *and is therefore recommended as a model*.*’* [14, 15]. Good practices are practices that are developed locally and with which health care professionals have good experience, but are not evidence-based and therefore not added to (inter)national guidelines [14, 15]. As a result, these practices are not distributed and applied nationally, such as the recommendations from (inter)national guidelines. Although not evidence-based, good practices can have additional advantages and are therefore worthwhile exploring. Moreover, local good practices for dialysis education and treatment could potentially explain the previously mentioned practice variation.

An overview regarding these good practices is lacking in current published literature. Thus, we performed a scoping review to identify and summarize the available literature describing good practices for dialysis education, treatment, and electronic health (eHealth).

## Methods

### Search strategy and selection criteria

Embase, Pubmed, the Cochrane Library, CINAHL databases and Web of Science were searched for relevant articles using all synonyms for the words ‘kidney failure’, ‘dialysis’, and ‘good practice’ (Table 1).

**Table 1:** Search strings.

Database	Search
Embase	hemodialys*: ab,ti OR haemodialys*:ab,ti OR ’hemo-dialys*’:ab,ti OR ’haemo-dialys*’:ab,ti OR ’renal dialys*’:ab,ti OR ’dialysis near/3 modalit*’:ab,ti OR ’artificial kidney’:ab,ti OR ’peritoneal dialys*’:ab,ti OR ’peritoneum near/3 dialys*’:ab,ti OR ’end stage renal*’:ab,ti OR ’kidney disease’:ab,ti OR ’kidney failure’:ab,ti OR ’peritoneal dialysis’/exp OR ’hemodialysis’/exp OR ’kidney disease’/exp AND ’good practice*’:ab,ti OR ’best practice*’:ab,ti
Pubmed	(hemodialys*[Title/Abstract] OR haemodialys*[Title/Abstract] OR hemo-dialys*[Title/Abstract] OR haemo-dialys*[Title/Abstract] OR "renal dialys*"[Title/Abstract] OR "dialys modalit*"[Title/Abstract] OR "artificial kidney*"[Title/Abstract] OR "peritoneal dialys*"[Title/Abstract] OR "peritoneum dialys*"[Title/Abstract] OR "End-Stage Kidney*"[Title/Abstract] OR "End Stage Kidney*"[Title/Abstract] OR "End-Stage Renal*"[Title/Abstract] OR "End Stage Renal*"[Title/Abstract] OR "Kidney failure"[Title/Abstract] OR "Renal Failure"[Title/Abstract] OR ESRD[Title/Abstract]) OR (renal dialysis[MeSH Terms] OR artificial kidneys[MeSH Terms] OR chronic kidney failure[MeSH Terms] OR dialysis, peritoneal[MeSH Terms] OR hemodialysis, home[MeSH Terms] OR kidney failure[MeSH Terms]) AND (("Good practice*"[Title/Abstract] OR "Best practice*"[Title/Abstract]) OR best practices[MeSH Terms])
Cochrane	((hemodialys* OR haemodialys* OR hemo-dialys* OR haemo-dialys* OR ’renal dialys*’ OR ’dialys modalit*’ OR ’artificial kidney*’ OR ’peritoneal dialys*’ OR ’peritoneum dialys*’ OR ’end-stage renal*’ OR ’end stage renal*’ OR ’chronic kidney failure’ OR ’end-stage kidney*’ OR ’end stage kidney*’ OR ESRD OR ’renal failure’):ti,ab,kw) OR (MeSH descriptor: [Renal Dialysis] Explode all trees) OR (MeSH descriptor: [Kidneys, Artificial] Explode all trees) OR (MeSH descriptor: [Renal Insufficiency, Chronic] Explode all trees) AND (("good practice*" OR ‘best practice*’):ti,ab,kw) OR (MeSH descriptor: [Practice Guidelines as Topic] Explode all trees)
CINAHL	(TI “hemodialys*”) OR (TI “haemodialys*”) OR (TI “hemo-dialys*”) OR (TI “haemo-dialys*”) OR (TI "renal dialys*") OR (TI "dialys modalit*") OR (TI "artificial kidney*") OR (TI "peritoneal dialys*") OR (TI "peritoneum dialys*") OR (TI "End-Stage Kidney*") OR (TI "End Stage Kidney*") OR (TI "End-Stage Renal*") OR (TI "End Stage Renal*") OR (TI "Kidney Failure") OR (TI "Renal Failure") OR (TI “ESRD”) OR (AB “hemodialys*”) OR (AB “haemodialys*”) OR (AB “hemo-dialys*”) OR (AB “haemo-dialys*”) OR (AB "renal dialys*") OR (AB "dialys modalit*") OR (AB "artificial kidney*") OR (AB "peritoneal dialys*") OR (AB "peritoneum dialys*") OR (AB "End-Stage Kidney*") OR (AB "End Stage Kidney*") OR (AB "End-Stage Renal*") OR (AB "End Stage Renal*") OR (AB "Kidney Failure") OR (AB "Renal Failure") OR (AB “ESRD”) OR (MH "Renal Replacement Therapy+") OR (MH "Dialysis+") OR (MH "Renal Insufficiency+") OR (MH "Kidney, Artificial") AND (AB "good practice*") OR (AB "best practice*") OR (TI "good practice*") OR (TI "best practice*") OR (MH "Professional Practice, Theory-Based+") OR (MH "Professional Practice, Research-Based+") OR (MH "Practice Guidelines")
Web of Science	TS = (hemodialys* OR haemodialys* OR hemo-dialys* OR haemo-dialys* OR "renal dialys*" OR "dialys modalit*" OR "artificial kidney*" OR "peritoneal dialys*" OR "peritoneum dialys*" OR "End-Stage Kidney*" OR "End Stage Kidney*" OR "End-Stage Renal*" OR "End Stage Renal*" OR "Kidney Failure" OR "Renal Failure" OR ESRD) AND TS = ("good practice*" OR "best practice*")

After removal of duplicates, two authors (AES and SV) independently screened titles and abstracts. Articles were eligible for inclusion if they provided a thorough description of the content of a good practice regarding dialysis education, treatment or eHealth for adult patients. Articles of all study types were included, however articles that described a guideline, review or meta-analysis were subsequently excluded after being screened for additional references.

Articles were excluded if they referred to a practice already covered in (inter)national guidelines, or if they reported on implementation projects, diabetes mellitus care or exercise programs for dialysis patients. In addition, articles were excluded if no full text or only a published abstract was available or if they were written in a language other than English.

The remaining articles were read full text by two authors (AES and SV) and screened for additional references. Final inclusion was based on consensus between the two authors (AES and SV) based on the previously mentioned in- and exclusion-criteria. In case of disagreement, the opinion of a third author (ACA) was decisive.

### Data extraction

Data extraction was executed and checked by two authors (AES and SV). The included studies were structured according to the category to which the good practice was related. The following categories were used: dialysis education, dialysis treatment, and eHealth. After classifying the articles in the aforementioned categories, the following data were extracted: study design, number of participants investigated, good practice description, results, and study conclusion.

## Results

### Study selection

**Fig 1:**
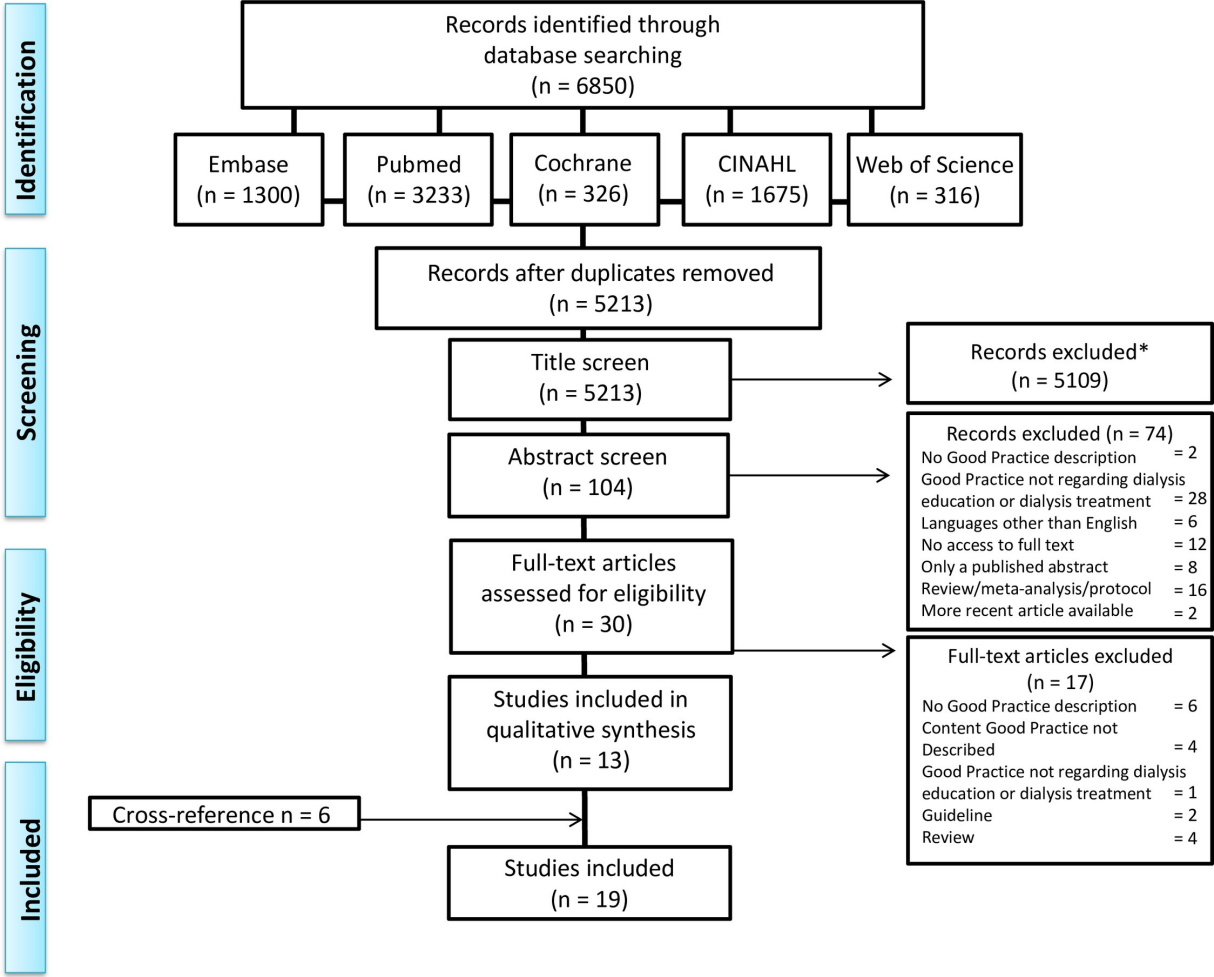
Selection flow diagram. * Exclusion criteria for title screen: No good practice regarding dialysis modality education/treatment or eHealth, implementation project, diabetes mellitus care or exercise program for dialysis patients, guideline, meta-analysis, protocol, review, and language other than English.

The initial literature search was performed on May 2, 2019, and last updated on January 12, 2021. Fig 1 provides an overview of the search. After removal of duplicates, the search provided 5,213 articles. Subsequently 5,109 articles were excluded based on the title and another 74 were excluded based on the abstract. The full-text of the remaining 30 articles was assessed for eligibility. In total, 17 articles were excluded for the following reasons: no good practice described [5, 16–20], content of the good practice not described [21–24], good practice not regarding dialysis education or dialysis treatment [25], articles describing a guideline [26, 27] or review [23, 28–30]. The remaining 13 articles were screened for additional references, resulting in 6 cross-references (Fig 1) [31–36]. No additional cross-references were found in the articles describing guidelines, reviews or meta-analyzes. So, in total 19 articles were included [31–49].

### Study characteristics

Characteristics of the 19 included articles are presented in Tables 2–4. Twelve articles described good practices for dialysis education (Table 2), three for dialysis treatment (Table 3), and four for eHealth (Table 4). All articles were published during the past 20 years and 47% of them came from the United States of America (USA). Most studies (58%) had a qualitative design, while the others were cohort studies (21%), case-control studies (11%), and randomized controlled trials (11%).

**Table 2:** Characteristics of studies on dialysis education.

Study, Country, Year	Study design, N	GP description	Results	Study conclusion
**Farina** [37] USA 2001	Quali-tative N = n.a.	Home visit for PD patients • Goals: • Visit prior to training: assess home environment, social and family dynamics. • Visit after training: assess application of PD procedures. • Components ○ Before visit: establish home visit policy, explain reason for visit to patient, secure directions to patient’s home, verify visit, review violence prevention, notify supervisor of planned visit. ○ During visit: review reason for visit, asses home (clean work area, adequate lighting, running water/soap, draft free room, free of pets, storage of supplies), survey equipment, compliance issues, family dynamics/adaptation skills, assessment of procedures/technique, medicines, provision of patient education, review of findings/recommendations. ○ After visit: review results with health care team and brainstorm to resolve problems, follow up on any issues that need to be resolved, track results of home visits over period of time, identify trends and opportunities for improvement.	n.a.	A home visit is a valuable tool and gives PD staff opportunity to monitor the environment where dialysis is performed.
**Figuei-redo** [38] 10 countries^a^2016	Quali-tative N = n.a.	PD training course syllabus^b^ • Day 1: Establish a report, describe goals and plan of the course, demonstrate steps of different procedures, assess patient learning styles/barriers, explain how learning will occur, introduce concepts of PD. • Day 2: Review goals, provide repeated supervised practice sessions of PD exchange and exit-site care with feedback from previous day, review concepts of asepsis, peritonitis, residual renal function, fluid balance, documentation, move from simple to more complex learning. • Day 3: Continue supervised procedure practice with feedback, review concepts through discussion and questions, introduce problem solving. • Day 4: Continue supervised procedure practice with feedback, including acknowledgment of skills mastered, review concepts through discussion and questions, continue to problem solve through “what if” scenarios. • Day 5: Review all previously presented concepts and practice all procedures until proficiency demonstrated.	n.a.	After completion of the PD training syllabus, the PD nurse will have provided education to a patient and/or caregiver such that the patient/caregiver has the required knowledge, skills and abilities to perform PD at home safely and effectively.
**Firanek** [39] USA 2013	Quali-tative N = n.a.	Features of successful nurse-led APD training • Setting and staff: dedicated staff members, training patients in clinic (home-like atmosphere, all necessary materials), home visit soon after training, then annually. • Training methods: five-step method; 1) overview (understand why learning procedure is necessary), 2) silent demonstration, 3) talking demonstration, 4) patient verbalizes each step procedure to demonstrate understanding/recall, 5) patient demonstrates procedure. • Educational documents: “less is more” in initial stages of patient training, 2 categories of educational documents (cycler, monitoring/troubleshooting). • Training structure: first CAPD training (2.5‒7 days for 3‒7.5 h/day), then APD training (1‒4 days for 3‒7.5 h/day). • APD training content: minimal educational materials, more complex topics at slower pace with simplified language and practical examples. • Delivery of APD training: verbal explanation of APD cycler, return demonstration by patient, instructions for washing hands/setting up supplies/starting cycler/preparing solution bags/loading set/connecting bags/priming and connecting patient/disconnecting patient/emergency disconnection/shut down cycler/clean and care for cycler, monthly education through verbal/written quizzes.	n.a.	Patient training programs should focus on basic and essential information patients need to master in order to dialyze successfully and safely at home. Clinics reinforced learning by several methods, including written quizzes to asses and document patient learning, and reviewing the quiz to reinforce learning and disseminate more information.
**Fortnum** [40] Australia and New Zealand 2015	Quali-tative N = 25	‘My Kidneys, My Choice’ decision aid for CKD patients focusing on SDM 1.‘My Kidneys’ (Deliberation talk): create awareness of need to make a decision for patient. 2.‘My Lifestyle’ (Deliberation/Choice talk): let patient acknowledge lifestyle impacts on options, educate about choices. 3.‘My Options’ (Choice/Option talk): start discussions/recap about treatment options. 4.‘My Choice’ (Decision talk): ask patient for readiness to make a decision, recentralize them in the SDM process, clarify they have understood options. 5.‘My Questions’: open page for patient to note questions, bring it back to subsequent appointments.	25 health professionals: • use aid: 11 times (±7.7) • mean score (1 ‘no help’ to 4 ‘very helpful’) ○ support understanding of options: 3.24±0.72 ○ assist understanding of patients’ priorities: 3.04±0.83 ○ support decision making: 3.17±0.72	The decision aid has the potential to improve decision making practice for CKD patients. Early acceptance is high.
**Lacson Jr** [31] USA 2011	Cohort N = 5600	Standardized predialysis treatment options education (TOP) for CKD patients • Goal: provide objective treatment options education to CKD patients and their families about renal transplant, ICHD, HHD, PD, conservative therapy. • Content: ○ Single group class session. ○ 30, 90, 180 days follow-up contact: 1) review treatment options, 2) inquire about each patient’s kidney function and dialysis access planning, 3) provide feedback to referring physician.	Adjusted OR for TOPs attendees vs. controls: • Select PD: 5.13 • (95%CI 3.58–7.35) • Start ICHD with fistula/ graft: 2.06 • (95%CI 1.88–2.26) • Mortality: 0.61 • (95%CI 0.50–0.74)	Attending TOP was associated with more frequent selection of PD, fewer tunneled HD catheters and lower mortality risk during the first 90 days of dialysis treatment.
**Luongo** [41] USA 2004	Quali-tative N = n.a.	Interview CKD patients for PD (Five-Step Approach) 1.Preparation: nurse explores questions regarding interview goals and competencies needed for interview, patient’s medical history/health care experience/culture/ background. 2.Environment: private room. 3.Special considerations: PD nurse must identify and manage variety of patient situations (geriatric patients, patients who do not speak or understand English, hearing or visually impaired, anxious or illiterate patients). 4.Interview: social history, home environment, language/education, physical limitations, general questions (e.g. previous experience, family member with CKD/RRT), financial issues, CKD/RRT education and information (e.g. review kidney function, PD), self-care issues. 5.Special concerns: pay attention to signs/situations that may predict future problems.	n.a.	The PD nurse has an important role in the patient’s health care experience and must use previous experience, clinical knowledge, and careful judgement to offer the future patient the correct information and support.
**Mandel** [42] USA 2017	Quali-tative N = n.a.	Serious Illness Conversation Guide for dialysis patients 1.Set up conversation: introduce idea/benefits, ask permission. 2.Assess illness understanding/information preferences. 3.Share prognosis: tailor information to patient preference, allow silence, explore emotion. 4.Explore key topics: goals, fears/worries, sources of strength, critical abilities, tradeoffs, family engagement/involvement. 5.Close the conversation: summarize what you’ve heard, make recommendation, affirm commitment to patient. 6.Document conversation.	n.a.	The Guide provides a tested, scalable structure for conducting serious illness conversations and assists in developing/adapting the care plan to ensure goal-consistent care.
**Manns** [32] Canada 2005	RCT N = 62	Educational intervention for CKD patients to promote self-care dialysis Phase 1: • 4 written patient manuals; 1 manual “Choosing the type of dialysis best suited to you”, 3 manuals on self-care dialysis (PD, HHD, self-care in-center HD). • 15-minute video “Choosing the type of dialysis best suited to you”. Phase 2: 90-minute small group interactive session involving 3‒6 patients, nephrologist, predialysis nurse.	Intervention group vs. standard care group: • Intention to start self-care dialysis: 82.1% vs. 50%, p = 0.015	A two-phase educational intervention can increase the proportion of patients who intend to initiate self-care dialysis.
**Martino** [33] Italy 2014	Case-control N = 188	Home visit program for PD patients • Home visits every 3 months between 2 visits PD center by skilled PD nurses. • Additional home visit in case of medical suggestions. • During home visit: • Nurse supervises environment of PD exchange, storage place of material, possible mistakes during procedures, compliance to pharmacological and dialysis therapy. • Nurse supports patients by suggesting possible solutions, reinforcing patient knowledge, and/or anticipating a medical visit to the PD center.	Home visit group vs. standard care group: • Treatment duration: 52 weeks vs. 48.8 weeks, p = 0.018 • Technique failure: 11.5% vs. 23.3%, p = 0.004 No difference for peritonitis and hospitalization rate.	The home visit program reduces technique failure and extends PD treatment.
**Michel** [43] USA 2005	Quali-tative N = n.a.	Conversations about prognosis with ESKD patients 1.Who to Tell; assess decision-making capacity of the patient, ask patient if he/she wants to hear prognosis and wants to participate in decision-making process. 2.When to Tell: early in course of progressive disease. 3.What to Tell: estimate of prognosis, life expectancy, likely QOL. 4.How to Tell: Method of Buckman and Kayson^c^ for breaking bad news.	n.a.	The approach should help discuss prog-nosis in a way that is sensitive to patients’ preferences in accor-dance with guideline recommendations.
**Wingard** [44] USA 2009	Case-control N = 1938	RightStart program for HD patients 3-month educational program coordinated by case manager (meeting 1‒2 times/week during 1^st^ month, every 1‒2 weeks for next months). • Intensive education focused on health self-management and rehabilitation. • Intensive nutritional counselling by dietitian, reinforced by case manager. • Interventions for achieving goals for anemia management, adequate dialysis dose, nutrition, reduction of catheter use, medication review, logistical and psychosocial support. • Collaboration with facility staff/medical director to ensure prompt and overall care.	RightStart vs. standard care patients: • Hospital days per patient year at 12 months: 7.2 vs. 10.5, p<0.001 • Mortality per 100 patient years at 12 months: 17 vs. 30, HR 0.59, p<0.001	The RightStart program decreases the number of hospital days and mortality for HD patients.
**Wu** [45] Taiwan 2009	Cohort N = 573	Multidisciplinary predialysis education (MPE) for CKD patients • Individual lectures CKD patients by nurse: ○ Stage 3 CKD (lecture every 3 months): healthy renal function, uremia presentation, risk factors and complications of renal progression, introduction to various RRTs (HD, PD, renal transplant). ○ Stage 4 CKD (lecture every 3 months): discussions on management CKD complications, indications of RRT, evaluation vascular/peritoneal access. ○ Stage 5 CKD (lecture every month): monitor timely RRT initiation, care of vas-cular/peritoneal access, dialysis-associated complications, registration for renal transplant waiting list. • All patients: dietary counselling (every 6 months).	MPE vs. standard care group (mean follow–up 11.7±0.9 months): • Requiring dialysis: 13.9% vs. 43.0%, adjusted HR 0.117 (95%CI 0.075–0.183) • All-cause mortality: 1.7% vs. 10.1%, adjusted HR 0.103 (95%CI 0.040–0.265)	MPE may decrease the incidence of dialysis and reduce mortality in late-stage CKD patients.

APD = automated peritoneal dialysis; CAPD = continuous ambulatory peritoneal dialysis; CI = confidence interval; CKD = chronic kidney disease; ESKD = end-stage kidney disease; GP = good practice; HD = hemodialysis; HHD = home hemodialysis; HR = hazard ratio; ICHD = in-center HD; MPE = Multidisciplinary predialysis education; N = number of people investigated; n.a. = not applicable; OR = odds ratio; PD = peritoneal dialysis; QOL = quality of life; RCT = randomized controlled trial; RRT = renal replacement therapy; SDM = shared decision making; TOP = treatment options education; USA = United States of America.

a. Australia, Brazil, Canada, China, Guatemala, Japan, Mexico, New Zealand, United Kingdom, and the United States.

b. Based on Knowles’s principles for adult education: 1) adults are internally motivated and self-directed; 2) adults bring life experiences and knowledge to learning experiences; 3) adults are goal-oriented; 4) adults are relevancy oriented; 5) adults are practical; 6) adult learners like to be respected [50].

c. 6-step approach of Buckman and Kayson for breaking bad news: 1) give news in person, in private, with sufficient time, without interruption; 2) find out what the patient’s preexistent knowledge is; 3) find out what the patient wants to know; 4) give a warning first, then provide a small amount of information in simple language at an appropriate pace for the patient; 5) respond to the patient’s feelings and concerns; 6) determine the next steps, identify sources of support, and make an early follow-up appointment [51].

**Table 3:** Characteristics of studies on dialysis treatment.

Study, Country, Year	Study design, N	GP description	Results	Study conclusion
**Abdel-Aal** [46] USA 2014	Quali-tative N = n.a.	PD catheter placement by interventional radiologists • Pre-procedure preparation: history/physical examination, stop anticoagulants 5 days before procedure, bowel preparation, fasting for 6h before procedure, pre-procedure antibiotics i.v., empty bladder, mark entry and exit site catheter. • Catheter placement procedure: patient in supine position, ultrasonography to determine safest entry and exit site, shave hair of abdomen and prep with antiseptic scrub, mild/moderate sedation, local anesthesia, ultrasound guided needle placement, fluoroscopic guided wire placement, placement catheter over wire, create exit site and catheter tunnel, fluoroscopic visualization to exclude kink in catheter and confirm proper location, testing of inflow and drainage catheter with 1L normal saline, incision closure and dressing.	n.a.	Placement of the PD catheter by IR is a cost-effective, minimally invasive alternative to traditional surgical placement.
**Craswell** [47] Australia 2020	Quali-tative N = n.a.	Practices for CVCs insertion / maintenance / removal Insertion: • Patient education prior to insertion: instructional, didactic approach. • Anatomical site selection and decision-making: preference for tunneled catheter, renal team responsible for decision-making regarding site/device. • Extent of training and de-skilling: lower skill level professional/insertion in different settings/after hours related to higher infection rates. • Patient cohort challenges: specific patient cohorts (e.g. ethnic background) affect infection rates.background) affect infection rates. Maintenance: • Assessment and monitoring for infection: nurses responsible. • Dressing practices and procedures to promote maintenance: nurses responsible. • Education about maintenance: patients and staff.background) affect infection rates. Removal: • Decision for removal: clinical decision, prompt in case of infection suspicion. • Catheter type dictates removal. • Complications of removal: uncommon (prolonged/difficult removal secondary to CVC being stuck, bleeding).	n.a.	This study demonstrates the perceived importance of the interdisciplinary team in the insertion, and management of dialysis CVCs and education of patients.
**Desai** [48] USA 2008	Quali-tative N = 342^a^	Good Practices to improve outcomes of dialysis centers (e.g. dialysis dose, anemia management) and survival in dialysis patients155 candidate practices, categorized in 8 major domains: 1. Facility characteristics and amenities 2. Facility-based health maintenance 3. Staff working climate 4. General dialysis care practices 5. Physician practices 6. Nursing practices 7. Technician practices 8. Miscellaneous practices	Outcomes related to: a. characteristics of multidisciplinary care conferences b. technician proficiency in protecting vascular access c. nurses training to provide education in fluid manage-ment, vascular access d. random/blinded audits of staff performance e. communication and teamwork among staff Disagreement about: 1. importance of facility-based health maintenance practices 2. optimal staffing ratios 4. frequency of dialysis-based physician visits 4. optimal frequency of multidisciplinary care	This study provides a “conceptual map” of candidate practices and highlights areas of general agreement and disagreement. These findings can help to provide targets for future research in quality improvement.

CVC = central venous catheter; GP = good practice; IR = interventional radiologists; i.v. = intravenous; N = number of people investigated; n.a. = not applicable; PD = peritoneal dialysis; USA = United States of America.

a. 342 respondents (nephrologists and nurses) for questionnaires regarding candidate good practices.

**Table 4:** Characteristics of studies on eHealth.

Study, Country, Year	Study design, N	GP description	Results	Study conclusion
**Kaldoudi** [34] Greece 2007	Quali-tative N = n.a.	Telehomecare for PD patients The PERKA^a^ service supports data collection and transmission from a patient’s home via phone or data networks to the PD clinic for monitoring and archiving: • PD data: PD method, PD prescription, actual PD daily treatment schema conducted (number of fluid exchanges, duration, solute type/volume, UF volume). • General biometric data and biosignals: body weight, blood pressure, heart rate, oxygen saturation, temperature (if required, ECG and glucose levels). • Free text or sound report and/or response to a structured questionnaire. The system contains a patient unit, a data collection unit, a web-based portal application, a database for patient data and a database for administrative data.	n.a.	The PERKA system enables telehome-care services for all PD patients.
**Li** [35] China 2014	RCT N = 135	Post-discharge nurse-led telephone support for PD patients^b^ Pre-discharge planning protocol (by nurse case manager): • Assessing patient’s physical, social, cognitive, emotional needs. • Conducting an individualized education program to strengthen and consolidate past learning experiences, clarify misconceptions and optimize health outcomes. Standardized 6-week post-discharge nurse-led telephone support: • Weekly telephone call for 6 consecutive weeks: first call within 72h after discharge to assess the patient’s status and to give advice. • Content of each telephone call is guided by the pre-discharge planning protocol and specific problems identified in pre-discharge assessment.	Effect of intervention: • QOL: better for symptom/problem (p = 0.01), work status (p = 0.02), staff encouragement (p = 0.01), patient satisfaction (p = 0.01), energy/fatigue (p = 0.02) • Less clinic visits (p = 0.039) • Blood chemistry and complication control: no effect.	Post-discharge nurse-led telephone support is helpful for some aspects of quality of life and reducing clinic visits of PD patients.
**Sicotte** [49] Canada 2011	Cohort N = 19	Telehemodialysis • Remote team of nephrologists and nurses provides specialized clinical supervision by tele-communicating with local care teams and patients. • Electronical health record that allowed the remote team to view laboratory results, vital signs and medication taken by the patient, and observe variables recorded by the dialysis machine in real-time as the treatments were administered. • Two organizational models: virtual patient rounds or telecase reviews with multidisciplinary teams.	Medication changes per month (follow-up 2 years): • Chibougamau community ○ Pre teleHD: 2.2±1.3 ○ Post teleHD: 1.8±1.5 • Chisasibi community ○ Pre teleHD: 8.1±5.4 ○ Post teleHD: 3.1±1.1 Intracommunity p = 0.01, intercommunity p = 0.002.No difference in number of HD sessions/month and transfers to hospital.	TeleHD can maintain the quality of care and can provide distant supervision while maintaining the level of care utilization. Different organizational models did not lead to differences in health condition or care utilization.
**Viglino** [36] Italy 2020	Cohort N = 107	Videodialysis (VD) for assisted PD patients Components: • Remote Station (patient’s home): video camera, monitor, microphone, technological box. • Control Station (center): webcam, computer, phone. • System connecting the 2 stations: real-time, high-quality audio-video transmission. Method of use: nursing staff can follow patients/caregivers during the phases: multi-user connection, acquisition and recording of dialysis parameters, performance of CAPD/APD procedure, filling dialysis sheets. VD sessions are also used for exit site care, assessing dialysis/clinical issues, checking adherence to pharmacological/ dietary therapy.	Peritonitis episodes: • VD patients: 1/84.2 months • Caregiver patients: 1/62.6 months. • Self-care patients: 1/45.2 months. Time to first peritonitis not different between groups.VD: 17.6% reduction in transfer from PD to HD due to reduced compliance/lack of caregiver availability.	VD-assisted PD is a reliable, safe system which requires no technological know-how and is easy to use when self-care is not possible due to physical, cognitive or psychological barriers.

APD = automated peritoneal dialysis; CAPD = continuous ambulatory peritoneal dialysis; ECG = electrocardiogram; GP = good practice; HD = hemodialysis; N = number of people investigated; n.a. = not applicable; PD = peritoneal dialysis; QOL = quality of life; RCT = randomized controlled trial; UF = ultrafiltration; VD = videodialysis.

a. The PERKA consortium consists of the School of Medicine in Democritus University of Thrace and two software companies (ALPHA Information Technology SA and VIDAVO Information Systems Inc.).

b. Unplanned admission to the Nephrology department and the PD catheter had to be in situ for at least 3 months.

### Dialysis education

Four of the twelve articles that described good practices for dialysis education, focused on providing objective predialysis education for CKD patients (Table 2) [31, 32, 40, 45]. Fortnum *et al*. [40] presented the ‘My Kidneys, My Choice’ decision aid, a patient-centered tool to support the education of CKD patients and promote shared decision making. Health care professionals found the decision aid to be helpful for understanding treatment options and patients’ priorities, and for supporting decision making.

Lacson Jr. *et al*. [31] initiated a standardized predialysis treatment options education program that consisted of education provided during a single group class session, followed by contacts after 30, 90, and 180 days during which treatment options were repeatedly discussed. Compared to controls, patients who followed the standardized education program were significantly more likely to choose PD (odds ratio (OR) 5.13) or to start in-center HD with a fistula or graft (OR 2.06), and had a lower mortality (OR 0.61) during the first 90 days of dialysis treatment [31].

Manns *et al*. [32] developed a two-phase patient-centered educational intervention, showing manuals and a video for self-care dialysis (i.e. PD, home HD, and self-care HD) in phase 1 and conducting a small group session in phase 2. The intervention significantly increased the proportion of patients who intended to initiate self-care dialysis (intervention group 82.1% vs. standard care group 50%).

Wu *et al*. [45] presented a multidisciplinary predialysis education program consisting of quarterly individual nurse-led lectures for CKD patients stage 3 and 4, while this was intensified to monthly lectures for CKD patients stage 5. Compared to controls, patients who followed the multidisciplinary education program had a significant lower risk of requiring dialysis (hazard ratio (HR) 0.117) and lower mortality (HR 0.103) after a mean follow-up of 11.7 months.

Five of the twelve articles that described good practices for dialysis education, focused on PD patients [33, 37–39, 41]. Luongo *et al*. [41] described a five-step approach (i.e. preparation, environment, special considerations, interview, and special concerns) for nurses to interview CKD patients who may choose PD as dialysis treatment. The goal of the interview was to reduce stress and anxiety in the patient and to promote shared decision making. Although this approach has not been tested, the authors concluded that it guides PD nurses in providing correct information to future PD patients without overwhelming them.

The qualitative studies of Figueiredo *et al*. [38] and Firanek *et al*. [39] focused on PD training. Figueiredo *et al*. [38] provided a detailed description of a 5-day PD training course, with an introduction on day 1, supervised procedure practice sessions on days 2 to 4, and a review of the provided information and check of the patient’s competence on day 5. The authors concluded that with this training course PD nurses ensure that the patient can perform PD safely and effectively. Firanek *et al*. [39] visited six centers to identify successful components of the PD training programs. Subsequently, they provided an overview of these successful components focused on setting and staff, training methods, educational documents, training structure, automated peritoneal dialysis (APD) training content, and delivery of APD training.

Successful home visit programs were described by Farina *et al*. [37] and Martino *et al*. [33]. The main similarities between the two programs were: assessment of the home where PD was performed, assessment of the PD procedure performed by the patient, and the patient’s compliance to pharmacological and dialysis therapy. While Farina *et al*. did not examine the effect of the intervention, Martino *et al*. reported that PD patients who received a home visit had a significantly longer PD duration (52 weeks) and a lower technique failure rate (11.5%) compared with controls (PD duration 48.8 weeks, technique failure rate 23.3%) [33].

The last three articles focused on an educational program for HD patients [44] and conversations with dialysis patients [42, 43]. Wingard *et al*. [44] described a 3-month educational program for HD patients that focused on health self-management, rehabilitation, nutritional counselling, and interventions for achieving goals such as anemia management, adequate dialysis dose, logistical, and psychosocial support. Compared to controls, patients who completed the program had significantly fewer hospitalization days per patient year (7.2 vs. 10.5) and a lower mortality (HR 0.59) after a maximum follow-up duration of 12 months. The authors concluded that the program not only reduced morbidity and mortality, but also increased job satisfaction for nurses.

Mandel *et al*. [42] described a 6-step guide for serious illness conversations with dialysis patients to discuss their prognosis. The guide consisted of the following steps: set up the conversation, assess the patient’s illness understanding, share the patient’s prognosis, explore key topics, close the conversation, and document the conversation. The article by Michel *et al*. [43] also described an approach for talking with dialysis patients about their prognosis based on four aspects: who to tell, when to tell, what to tell, and how to tell. The authors concluded that this approach can help discussing prognosis with dialysis patients, taking into account the patient’s preferences.

### Dialysis treatment

The three articles that described good practices for dialysis treatment were all qualitative studies (Table 3) [46–48]. Abdel-Aal *et al*. [46] provided a detailed description of the procedure for insertion of a PD catheter by interventional radiologists. Various aspects of pre-procedure preparation, such as bowel preparation and fasting, were discussed followed by a detailed explanation of the PD catheter insertion with explanatory photos. The procedure was described as a cost-effective and minimally invasive alternative to traditional surgical placement of a PD catheter.

Craswell *et al*. [47] described practices for insertion, maintenance, and removal of central venous catheters (CVCs) for HD. The practices for insertion consisted of patient education for insertion, anatomical site selection and decision-making, and training. The practices for maintenance consisted of education, dressing practices, and assessment and monitoring for infection. The practices for removal consisted of the decision for removal and complications of removal. The authors concluded that an interdisciplinary team is very important for patient education and catheter care.

Desai *et al*. [48] reported 155 good practices that could potentially improve outcomes of dialysis centers, such as dialysis dose and anemia management, and overall survival in dialysis patients. The 155 good practices were divided into the following domains: facility characteristics and amenities, facility-based health maintenance, staff working climate, general dialysis care practices, physician practices, nursing practices, technician practices, and miscellaneous practices. Through a survey among 342 respondents, a top 30 of good practices that had the most impact on overall outcomes in dialysis was compiled. The majority of the top 30 good practices focused on conducting a successful multidisciplinary team meeting, performing audits, training nurses, reviewing the performance of health care professionals, and enhancing communication and teamwork.

### eHealth

Four articles described good practices for eHealth, one of which focused on HD [49] and three on PD (Table 4) [34–36]. The qualitative article on PD by Kaldoudi *et al*. [34] described the components of an eHealth system by which data could be collected such as PD method, prescription, body weight and hearth rate. Viglino *et al*. [36] described an eHealth system which led to a reduction in peritonitis episodes and a 17.6% reduction in the number of transfers from PD to HD because reduced compliance or lack of availability of a caregiver was no longer an issue. The authors concluded that this system can be a valuable tool for increasing the number of PD patients.

While Kaldoudi *et al*. [34] and Viglino *et al*. [36] focused more on the technical aspects of eHealth systems for PD patients, Li *et al*. [35] conducted a randomized controlled trial to investigate the effect of post-discharge telephone support for PD patients. Patients were included if they performed PD for a minimum of 3 months and were admitted to a nephrology department. The control group received routine care, while patients in the intervention group were visited by a nurse who assessed their needs and provided individualized education. After discharge from the hospital, the nurse called the patients from the intervention group every week during a period of 6 weeks to assess their status and to give advice. This approach led to a significant improvement of several health-related quality of life domains (e.g. symptoms, energy, work status) and a reduction in the number of hospital visits.

Finally, Sicotte *et al*. [49] reported two eHealth models for in-center HD patients: virtual patients rounds and telecase reviews with a multidisciplinary team. During the virtual patient rounds, a remote nephrologist and nurse had contact with a patient and his/her nurse at the dialysis center. During the telecase review, a remote nephrologist and nurse had contact with the general practitioners and nurses at the dialysis center via videoconference, without the patient being present. Both models led to a significant reduction in the number of medication changes per month during a follow-up of 2 years. The authors concluded that eHealth can provide distant supervision which improves the level of care utilization.

## Discussion

This scoping review identifies 19 articles with good practices that could be used in addition to guidelines. The twelve articles with good practices for dialysis education endorse the importance of providing complete and objective predialysis education to CKD patients, assisting PD patients in performing PD adequately, educating HD patients on self-management, and talking with dialysis patients in general about their prognosis. The three articles with good practices for dialysis treatment provide practices regarding dialysis access devices and numerous candidate good practices for dialysis centers. Finally, eHealth is useful for HD and PD and affects both quality of care and health-related quality of life.

Good practices are locally implemented practices with which health care professionals have good experience, but which are not necessarily evidence-based [14, 15]. Therefore, they are generally not added to (inter)national guidelines. For dialysis treatment, there are many guidelines with proven treatment methods, while guidelines for dialysis education are scarce [10, 52]. This probably explains why we have found many good practices for dialysis education and only a few for dialysis treatment.

Six of the 12 articles regarding dialysis education report a positive effect of the described good practice(s) [31–33, 40, 44, 45]. Complete and objective education to CKD patients by a multidisciplinary team decreases the dialysis incidence and mortality [45]. Moreover, it increases the use of home dialysis [31, 32]. The European Renal Best Practice (ERBP) Advisory Board also underscores complete and objective education to enable CKD patients to choose a dialysis modality that is most suitable for them [5]. Another useful good practice is a decision aid for CKD patients, which supports the shared decision making process according to health care professionals [40]. A Cochrane review, describing 105 decision aids for patients facing various treatment or screening decisions, also states that decision aids increase participants’ knowledge, decrease decisional conflicts, and facilitate active participation in decision making [53]. However, the review includes no decision aids specifically for nephrological care. A randomized study among 133 CKD patients concludes that an online decision aid can improve knowledge and decrease decisional conflict and uncertainty about choice of dialysis treatment [54]. So, decision aids are important for use during dialysis education.

A home visit also seems to be a very relevant tool for PD education, since Martino *et al*. [33] report that their home visit reduces technique failure and extends PD treatment. The positive effect of a home visit is also found in a French study of 359 patients on assisted PD, which found that it increases the probability of patients remaining peritonitis free from 33.9% to 50.8% at 3 years (p = 0.028) [55]. Home visits conducted in two other studies, with the aim of providing dialysis education for CKD patients, result in a higher probability for patients to receive home dialysis [56, 57]. So, home visits seem to be important not only for PD patients, but also for CKD patients who have yet to make a treatment choice.

The articles regarding dialysis treatment provide guidance on PD catheter placement by interventional radiologists and the insertion, maintenance, and removal of CVCs [46, 47]. The International Society for Peritoneal Dialysis (ISPD) guideline on peritoneal dialysis access only briefly mentions image-guided percutaneous PD catheter placement [58], so the procedure described by Abdel-Aal *et al*. can be a relevant addition [46]. The (inter)national guidelines for CVCs also describe insertion, maintenance, and removal practices [59–61], however only the most recent guideline [62] underscores the importance of patient education as Craswell *et al*. did [47]. Finally, the 155 candidate good practices reported by Desai *et al*. could lead to general quality improvement of dialysis care [48].

The articles regarding eHealth show that this good practice improves quality of care for HD patients [49], quality of life for PD patients [35], and reduces the number of peritonitis episodes [36]. In 2017, Rosner *et al*. [63] conducted a review on the use of eHealth in the care for dialysis patients. They found 19 articles describing mostly small, single-center studies published between 1999 and 2017, 13 articles for PD and 6 articles for HD. Most of the articles used video conferencing, remote monitoring, or monthly visits with physical examination (e.g. electronic stethoscopes) using eHealth as technology. All articles report positive results of their eHealth system on various outcomes such as patient independence, quality of life, and hospitalization. Rosner *et al*. conclude that there still is a lack of evidence regarding the use of eHealth, however they mention possible benefits for example increased uptake and acceptance of home dialysis, treatment monitoring in the home environment, improved patient satisfaction, and potential for cost savings [63]. In the current time with the coronavirus disease 2019 (COVID-19) pandemic, eHealth may play an important role through, for example, video conferences and remote patient monitoring [64–66].

Our review has several limitations. First, there is a probability that we have not identified all articles describing good practices. This is partly because many articles do not label their practice as ‘good practice’, making them less likely to appear in the search. However, by also using ‘best practice’ and ‘practice guidelines’ as a search topic, we believe that we have attenuated this problem. Second, most of the studies are qualitative in nature and describe no results, making it impossible to determine an effect of the described good practices. Finally, most of the studies that described results investigate a small number of patients and report on different outcomes, making mutual comparison impossible.

In conclusion, our scoping review identifies 19 articles describing good practices and their results for dialysis education, dialysis treatment, and eHealth. These good practices could be valuable in addition to guidelines for increasing shared-decision making in predialysis education, using patients’ contribution in the implementation of their dialysis treatment, and advanced care planning. Good practices can inspire and support health care professionals to change their practices and this could possibly help to improve outcomes and quality of life for CKD and dialysis patients. Additional research on good practices could be useful to identify more good practices and determine the impact of these practices on CKD and dialysis patients.
